# The Composite Homeostatic Wave: A Soliton-Based Framework for Understanding Consciousness Loss and Systemic Pathology in Aneurysmal Subarachnoid Hemorrhage

**DOI:** 10.7759/cureus.108062

**Published:** 2026-04-30

**Authors:** Eric Whitney

**Affiliations:** 1 Neurosurgery, Arrowhead Regional Medical Center, Colton, USA

**Keywords:** aneurysm rupture pathology, cerebrospinal fluid acoustics, consciousness loss mechanism, cortical spreading depolarization, homeostatic wave system, leptomeningeal membrane network, neurogenic stunned myocardium, soliton nerve propagation, subarachnoid hemorrhage, wave coherence failure

## Abstract

The systemic pathology following aneurysmal subarachnoid hemorrhage (SAH), including loss of consciousness, neurogenic stunned myocardium, cardiac arrhythmias, pulmonary edema, gastrointestinal dysmotility, and prolonged circadian and emotional sequelae, is conventionally attributed to a sympathetic catecholamine surge from hypothalamic irritation. This explanation, while partially valid, does not fully account for the variability in consciousness loss among patients with similar hemorrhage volumes, the scope of multi-organ involvement, or the persistence of sequelae long after catecholamine normalization.

This paper proposes a qualitative conceptual framework: the human body may be usefully modeled as a composite homeostatic wave system--a coupled network of electromagnetic, mechanical, and thermodynamic oscillations whose organized dynamics constitute physiological function. Drawing on the Heimburg-Jackson soliton model, which treats action potentials as adiabatic electromechanical density waves in lipid membranes, I argue that both cerebrospinal fluid (CSF) and the leptomeningeal membranes through which it permeates function as critical components of the neural wave-propagation system. Blood entering the subarachnoid space simultaneously alters CSF acoustic impedance and directly disrupts the leptomeningeal connective tissue network, a continuous collagenous membrane system with piezoelectric properties demonstrated in vitro that extends from the cranial meninges through the spinal nerve root sheaths and may communicate with the peripheral fascial network. This dual disruption of both the fluid medium and the membrane substrate produces global wave-coherence failure, offering a unified explanation for the multi-system pathology observed after SAH. The term "soliton-based" in this paper's title refers specifically to the Heimburg-Jackson model as one mechanistic foundation for the neural wave-propagation component; the composite homeostatic wave framework is broader, encompassing electromagnetic, mechanical, and thermodynamic oscillatory modes whose disruption collectively produces the multi-system pathology described here.

This framework generates five testable predictions designed to distinguish it from the standard catecholamine-surge model and to motivate future mathematical formalization. If confirmed, these predictions may inform novel therapeutic strategies focused on restoring the wave-propagation environment rather than solely managing downstream consequences.

## Introduction and background

The clinical problem

Aneurysmal subarachnoid hemorrhage (SAH) produces a distinctive constellation of systemic pathology that extends far beyond the mechanical effects of intracranial blood. Approximately half of patients lose consciousness at the moment of rupture, some regaining awareness within minutes, while others progress to coma [1]. The systemic consequences include neurogenic stunned myocardium (NSM) with electrocardiographic changes mimicking acute coronary syndrome [2], pulmonary edema, gastrointestinal dysmotility, circadian rhythm disruption persisting for weeks to months, and emotional lability that can endure for years.

The standard explanation attributes these multi-organ effects to a sympathetic catecholamine surge triggered by hypothalamic irritation from subarachnoid blood [2,3]. While catecholamine elevation is well-documented and contributes to cardiac pathology, this model does not fully explain three observations: the variability in consciousness loss among patients with similar hemorrhage volumes, the scope of involvement across organ systems with different autonomic innervation patterns, and the persistence of sequelae long after catecholamine levels normalize.

These gaps suggest that an additional mechanism may contribute to SAH pathology, one operating through the alteration of the physical medium and membrane structures through which physiological signals propagate.

The soliton model of nerve propagation

The Hodgkin-Huxley model treats the action potential as a purely electrical phenomenon, a voltage spike propagated by sequential ion channel gating [4]. This model, while enormously successful, leaves several observations unexplained: the reversible heat exchange during action potentials (the nerve heats during depolarization and cools during repolarization, with near-zero net heat dissipation) [5,6], the mechanical displacement of the nerve fiber that accompanies the electrical signal [7], and the observation that anesthetic potency correlates with lipid solubility across structurally diverse agents spanning several orders of magnitude (the Meyer-Overton rule) [8]. It should be noted that the Meyer-Overton correlation is now considered incomplete: specific molecular targets for anesthetics have been identified, including GABA-A receptors, glycine receptors, and two-pore-domain potassium channels [9]. However, the identification of these receptor targets has not explained why the thermodynamic correlation persists across such chemically diverse compounds, an observation that remains mechanistically unaccounted for in purely receptor-specific models.

Heimburg and Jackson proposed that the nerve impulse is an adiabatic electromechanical density wave, a soliton-propagating through the lipid membrane near its phase transition temperature [10,11]. In this model, the nerve impulse is fundamentally a thermodynamic phenomenon--a localized compression wave that carries electrical, mechanical, and thermal changes simultaneously as a coupled package. Subsequent work has extended the model theoretically [12,13] and has shown that these electromechanical pulses can be experimentally induced in lipid monolayers and biological membranes [14].

The soliton model remains a minority view in neuroscience and has not fully reconciled its predictions with the well-documented contributions of voltage-gated ion channels to signal propagation. Nevertheless, its core predictions--reversible heat exchange, mechanical displacement, and thermodynamic anesthetic mechanism--are consistent with experimental observations that the Hodgkin-Huxley model does not naturally explain [15].

## Review

The composite homeostatic wave concept

Clinical medicine already recognizes the body as an oscillatory system. Every organ is monitored by its wave characteristics: electroencephalography (EEG) for the brain, electrocardiography for the heart, electromyography for skeletal muscle, and respiratory waveforms for the lungs. Health is defined by the organized characteristics of these waves (normal sinus rhythm, organized EEG patterns, and rhythmic respiratory cycles), and pathology is diagnosed by their disruption (arrhythmias, seizure activity, and irregular breathing patterns).

This paper argues that these clinical observations reflect a deeper physical reality: the human body operates as a composite homeostatic wave system, a coupled network of electromagnetic, mechanical, and thermodynamic oscillations whose organized dynamics constitute physiological function. Homeostasis, in this framework, is not merely a set of chemical equilibria but the maintenance of organized wave coherence across multiple coupled oscillatory modes.

Multiple oscillatory modes coexist and interact. Heart rate variability reflects cardiac-autonomic coupling, with reduced variability associated with increased cardiovascular morbidity and mortality [16]. The perturbational complexity index measures the organized complexity of cortical responses to electromagnetic stimulation, distinguishing conscious from unconscious states [17]. Circadian disruption produces measurable cardiovascular and metabolic pathology [18]. These are not metaphors; they are empirical observations of coupled oscillatory behavior in biological systems.

CSF as a wave-propagation medium

Cerebrospinal fluid (CSF) is not merely a cushion or waste-clearance system. It is a fluid medium with specific acoustic properties: a sound velocity of approximately 1,500 m/s and acoustic impedance characteristics that vary with protein content and cellular composition [19]. Computational hydrodynamic modeling of the spinal CSF cavity suggests that CSF pulse waves may propagate at velocities ranging from 13 to 124.5 m/s, depending on the propagation mode and coupling to surrounding structures [20]. Empirical MRI phase-contrast measurement of the CSF velocity wave speed in the cervical spinal canal has yielded a mean of 4.6 m/s (SD 1.7 m/s) using cardiac-gated MR velocimetry in a pilot study of three subjects [21], establishing that the CSF compartment functions as an active mechanical wave-propagation medium, distinct from bulk flow. While these velocities describe mechanical pressure-wave transmission rather than neural signaling per se, they confirm that the CSF compartment's transmission characteristics are determined by its physical properties and, therefore, altered when those properties change. The CSF pulse waveform contains specific frequency components that reflect intracranial structural parameters-arterial pulsation, intracranial compliance, and venous outflow dynamics [22].

From a wave-propagation perspective, CSF provides the medium through which mechanical (pressure) waves couple the cerebrovascular system to the neural tissue. Changes in CSF composition alter the acoustic impedance of this medium, modifying wave-propagation characteristics throughout the system.

The leptomeningeal membrane network

The leptomeninges, the arachnoid and pia mater, are not passive wrappings. They form a continuous connective tissue network composed primarily of type I collagen fibers with an average fiber width of approximately 30 μm and a volume fraction of approximately 26% in the subarachnoid space [23,24]. This trabecular network partitions the subarachnoid space into functional compartments and creates a three-dimensional structural matrix through which CSF flows. The recently proposed subarachnoid lymphatic-like membrane (SLYM) may represent an additional membranous layer dividing the subarachnoid space into functional compartments [25], though this finding remains contested and awaits independent replication.

Critically, this membrane network is physically continuous. The leptomeninges extend from the cranial vault through the spinal canal, and at each spinal nerve root, the meningeal connective tissue continues into the epineurium and perineurium of the peripheral nerves [26]. Collagen exhibits piezoelectric properties demonstrated in vitro [27,28]: mechanical deformation generates electrical potentials, and conversely, electrical fields produce mechanical strain. Langevin has proposed that the connective tissue system may function as a body-wide signaling network [29], and fibroblasts within fascia have been shown to respond to mechanical stimulation with intracellular signaling cascades in cell-culture models [30]. Whether these properties constitute a functional signaling system at physiological scales in vivo remains unproven.

SAH as a wave-system disruption

When an intracranial aneurysm ruptures, arterial blood enters the subarachnoid space--the CSF-filled compartment bounded by the leptomeningeal membrane network. From a wave-propagation perspective, this event simultaneously transforms the propagation medium and damages the membrane substrate.

What does blood do to the wave system?

Blood in CSF changes nearly every physical property relevant to wave propagation. Whole blood has a density approximately 6% greater than CSF (1,060 versus 1,007 kg/m³) [31]. Its viscosity is dramatically higher; plasma proteins, including fibrinogen, contribute substantially to viscosity elevation compared to CSF [32]. Red blood cells introduce particulate scattering. Hemoglobin and its degradation products alter optical absorption across the ultraviolet-visible spectrum. And the ionic composition of blood differs from CSF, altering local electrical conductivity.

The acoustic impedance changes affect mechanical wave modes (CSF pressure waves, pulse waves). The conductivity changes from blood's ionic content alter volume-conducted electrical fields--the near-field phenomena that EEG records. The optical absorption changes would affect any biophotonic emission in the ultraviolet-visible range; should such emission prove to exist, biophotonic emission represents an emerging area with preliminary evidence but no confirmed functional role in SAH pathophysiology [33,34]. And the membrane-level disruption--oxidative damage, inflammatory alteration, and mechanical deformation--would alter the thermodynamic conditions required for soliton propagation in the membranes themselves. Critically, this transformation occurs simultaneously across the entire CSF-bathed surface of the nervous system.

The wave-disruption model of consciousness loss

Under the composite homeostatic wave framework, the loss of consciousness in SAH involves wave-coherence disruption operating concurrently with, and potentially preceding, the hemodynamic cascade. The organized oscillatory dynamics that constitute cortical function are disrupted because both the medium through which they propagate and the membrane structures in which they are embedded have been acutely and globally altered.

This model better explains the variability in consciousness loss than the intracranial pressure (ICP)-cerebral perfusion pressure (CPP) model alone. If consciousness depends on wave coherence rather than solely on perfusion pressure, then the distribution of blood relative to critical oscillatory coupling structures matters more than total volume. Blood in the basal cisterns--surrounding the brainstem reticular activating system, hypothalamus, and cranial nerve exit zones, and directly contacting the dense leptomeningeal trabecular networks that partition these cisterns [23]--would disrupt more critical coupling nodes than convexity blood of equal volume. This is consistent with clinical observation that cisternal blood distribution (as captured by the Hijdra sum score) predicts outcomes differently than simple volumetric measures.

The systemic cascade as wave propagation

The multi-organ pathology following SAH-NSM, pulmonary edema, gastrointestinal dysmotility, circadian disruption, and emotional lability has been attributed to catecholamine-mediated sympathetic hyperactivation [2,3]. This explanation has empirical support: serum catecholamine levels are elevated, and myocardial biopsy shows contraction band necrosis consistent with catecholamine toxicity [35]. Metabolic and neuroinflammatory mechanisms, including spreading cortical oligemia, microglial activation, and neuroinflammatory cascades, also contribute to delayed injury after SAH. The wave-disruption model does not compete with these mechanisms; it proposes a physical disruption layer that may precede and amplify metabolic injury by altering the propagation environment in which these processes unfold. However, several features are not well explained by catecholamines alone.

NSM after SAH produces electrocardiographic changes, troponin elevation, and wall motion abnormalities that mimic acute coronary syndrome so closely that cardiologists sometimes cannot distinguish them [2]. Nuclear scintigraphic studies reveal that subclinical metabolic myocardial alterations occur in the vast majority of hemodynamically stable SAH patients, even when conventional cardiac evaluation is entirely normal, suggesting a widespread neurometabolic disruption that extends beyond clinically apparent cases [36]. Circadian rhythm disruption persists for weeks to months long after catecholamines have normalized. Emotional lability and personality changes can persist for years.

The wave-disruption model offers a different and complementary account. If the body operates as a coupled oscillatory system, then disruption of the central oscillatory hub (the CNS, with its CSF medium and leptomeningeal membrane network now contaminated) would propagate through all coupled pathways: via the sympathetic chain to the heart, via the vagus to the gut, via hypothalamic-pituitary coupling to the circadian system, and via limbic-cortical coupling to emotional regulation. The leptomeningeal-fascial membrane continuum provides an additional propagation pathway-structural disruption at the meningeal level could alter the mechanical and piezoelectric signaling properties of the connected fascial network, contributing to the body-wide nature of the systemic response. This pathway is speculative; whether the piezoelectric and mechanical properties demonstrated for collagen in vitro translate to a functional signaling system in vivo remains unproven, and the framework’s core predictions do not require it. The catecholamine surge, in this framework, is one component of the wave-disruption cascade rather than its sole explanation.

Relationship to cortical spreading depolarization

Any discussion of wave phenomena in SAH must engage with the extensive literature on cortical spreading depolarization (CSD). CSDs are self-propagating waves of neuronal and glial mass depolarization that occur with high incidence after SAH and are strongly associated with worse tissue injury and clinical outcomes [37,38]. They propagate across the cortical surface at 2-5 mm/min, producing transient failure of brain ion homeostasis, and are now recognized as a major mechanism of delayed cerebral ischemia [39]. The volume of SAH directly correlates with CSD occurrence, and hemoglobin degradation products have been shown to trigger CSDs experimentally [38].

CSD represents a specific pathological wave phenomenon, a wave of depolarization, within the cortex. The composite homeostatic wave model addresses a different and broader question: the disruption of the body's normal oscillatory coherence across multiple organ systems through alteration of the propagation system (CSF + membranes) rather than through the generation of a single pathological wave type. However, the two frameworks are complementary rather than competing. CSDs may themselves be a consequence of the medium disruption this paper describes, a cortical response to the altered thermodynamic, chemical, and mechanical environment produced by blood in the subarachnoid space. The observation that hemoglobin degradation products trigger CSDs [38] is precisely what the wave-disruption model would predict: the altered medium generates pathological wave behavior. Furthermore, the finding that even focal accumulations of subarachnoid blood are sufficient to trigger CSD clusters [38] supports the concept that the propagation environment, not just perfusion status, determines wave behavior in the injured brain.

The composite homeostatic wave framework offers a specific mechanistic account of why CSD produces ischemic injury preferentially in the post-hemorrhagic brain. Under normal physiological conditions, CSD is accompanied by vasodilation and increased cerebral blood flow--a neurovascular coupling response that limits tissue injury [37]. In the SAH environment, this coupling inverts: CSD triggers vasoconstriction rather than vasodilation, producing the spreading cortical oligemia associated with delayed infarction [38,39]. The composite homeostatic wave framework provides a candidate explanation for this inversion. Blood in the subarachnoid space has altered the acoustic, chemical, and thermodynamic properties of the medium in which both the depolarization wave and the vascular response must propagate. The altered propagation environment does not merely permit CSD; it transforms its vascular consequences. This framing predicts that the probability of inverse neurovascular coupling during CSD should correlate with the degree of CSF medium disruption, measurable through the acoustic impedance parameters proposed in Prediction 2, rather than simply with time from hemorrhage or CSD cluster density. If correct, this would identify patients at highest risk of CSD-mediated infarction on the basis of their CSF medium properties, not merely their electrocorticographic burden.

Testable predictions

A hypothesis that cannot be distinguished from the standard model is not useful. The composite homeostatic wave model makes specific predictions that differ from the catecholamine-surge model.

Blood distribution should predict cardiac complications better than catecholamine levels

If the wave-disruption model is correct, then the distribution of subarachnoid blood relative to key oscillatory coupling structures (brainstem, hypothalamus, and basal cisterns) should predict cardiac complications more strongly than peak serum catecholamine or norepinephrine metabolite levels. The critical analysis would stratify by cisternal versus convexity blood distribution using volumetric quantification (Hijdra sum score), correlating against both catecholamine levels and cardiac outcomes. Existing data suggest that the Hunt-Hess grade, which reflects neurological status rather than blood volume alone, predicts cardiac complications [2], consistent with a model where the functional disruption of oscillatory coupling, not simply the hemorrhage mass, drives systemic pathology. I note that existing grading scales, including the modified Fisher scale and Hijdra sum score, were developed to predict vasospasm and delayed cerebral ischemia, not extracranial systemic complications. The novel element of this prediction is the specific correlational analysis: stratifying by cisternal versus convexity blood distribution and correlating against both catecholamine levels and cardiac outcomes simultaneously. To my knowledge, this analysis has not been performed.

CSF acoustic and optical properties should change measurably

Direct measurement of CSF acoustic impedance at diagnostic ultrasound frequencies (1-10 MHz range) and optical absorption from serial ventriculostomy samples should reveal quantifiable differences between bloody and clear CSF. Specifically, I predict that acoustic impedance deviation from normal CSF baseline values should correlate with spectrophotometric xanthochromia grading and with the temporal progression and resolution of specific systemic symptoms, including cardiac rhythm abnormalities, gastrointestinal dysmotility, and circadian disruption. A dose-response relationship between impedance deviation magnitude and systemic symptom severity would constitute strong confirmatory evidence for the wave-disruption model. The technology for acoustic impedance measurement of small fluid volumes exists and could be implemented at the bedside using existing ultrasound transducer technology.

ICP waveform morphology should predict systemic complications

If the wave-medium is disrupted by blood, ICP waveform morphology should show specific alterations, not just elevated mean ICP, but also changes in harmonic content, P1/P2 ratio dynamics, and waveform entropy that reflect altered wave-propagation properties. The prediction is that ICP waveform complexity measures should correlate with systemic complications independently of mean ICP level. Cardoso (1983) demonstrated that specific ICP waveform components reflect different intracranial structural parameters [22], but no study has correlated ICP waveform complexity with extra-cranial complications.

Severity of persistent complications should correlate with blood duration, not peak catecholamines

If the wave-disruption model contributes, then persistent complications should correlate more strongly with the duration and distribution of subarachnoid blood (which determines how long the medium remains disrupted) than with peak catecholamine values. These are empirically distinguishable predictions and could be tested retrospectively in existing SAH databases that include serial catecholamine measurements and imaging.

Accelerated CSF clearance should improve systemic outcomes beyond ICP reduction

The most direct therapeutic prediction: if blood in the CSF disrupts wave coherence, then accelerated clearance of blood from the subarachnoid space should improve systemic outcomes beyond what ICP reduction alone predicts. The glymphatic system--the perivascular CSF circulation pathway through which the brain clears metabolic waste--provides a mechanistic substrate for this prediction: subarachnoid blood impairs glymphatic flow through both physical obstruction of perivascular spaces and altered CSF rheology, amplifying medium disruption and delaying the restoration of normal propagation conditions [40]. Lumbar drainage and intrathecal thrombolysis are already used to reduce vasospasm risk, but no study has examined whether these interventions specifically improve cardiac, gastrointestinal, circadian, or emotional outcomes when controlled for ICP.

Connections to related frameworks

The soliton model explains anesthesia as a thermodynamic effect: anesthetic agents dissolve in the lipid membrane and shift its phase transition temperature [11]. Under the composite homeostatic wave framework, general anesthesia represents a pharmacological disruption of wave coherence. The loss of consciousness in SAH may operate through a parallel mechanism: instead of the membrane's properties being altered pharmacologically, both the surrounding medium and the connected membrane network are altered physically. Both produce wave-coherence disruption; the mechanisms differ, but the result--loss of consciousness--is analogous.

This parallel suggests that SAH patients with residual subarachnoid blood may exhibit altered anesthetic sensitivity compared to patients with clear CSF. However, the clinical confounders in SAH patients (cerebral edema, elevated ICP, altered cerebral blood flow, hemodynamic instability, medication interactions) make direct clinical testing impractical. An in vitro or ex vivo approach--measuring soliton propagation characteristics or anesthetic thresholds in nerve preparations bathed in bloody versus clear artificial CSF--would provide a more tractable experimental model.

The framework is additionally compatible with, but does not require, contributions from quantum biological mechanisms such as orchestrated objective reduction [41] or biophotonic emission in neural tissue [33,34]. These remain speculative and contested--Orch OR in particular faces substantial objections regarding thermal decoherence at biological temperatures--and the present paper’s five testable predictions are entirely classical, requiring no quantum coherence or biophotonic processes.

Limitations

Several important limitations must be acknowledged. First, the soliton model of nerve propagation, while published in reputable journals and actively researched, remains a minority view in neuroscience. The composite homeostatic wave framework is built partly on this foundation, and to the extent the soliton model is incorrect, the mechanistic specificity of the argument is weakened. However, the broader observations--that the body operates as a coupled oscillatory system, that CSF is a wave-propagation medium, that leptomeningeal membranes possess electrical properties, and that blood in CSF alters all of these--do not require the soliton model. These observations are valid within the standard electromagnetic and biomechanical frameworks.

Second, the term "composite homeostatic wave" currently lacks mathematical formalization. To be fully rigorous, the wave modes must be specified, the coupling mechanisms formalized, and the dispersion relations derived. This paper provides the qualitative framework and testable predictions; the mathematical treatment is a necessary next step requiring collaboration between neurosurgeons, biophysicists, and applied mathematicians. Specifically, a rigorous treatment would need to specify the coupling tensor between the electromagnetic, mechanical, and thermodynamic modes; derive the dispersion relations for the composite system; and establish whether the coupled modes support stable soliton-like solutions under physiologically realistic parameter values.

Third, the piezoelectric and semiconductive properties of collagen have been demonstrated in vitro [27,28], and whether they function as a signaling system in vivo at physiologically relevant scales remains unproven. The fascial communication hypothesis (Langevin 2006) is plausible and has been published, but not yet established. This paper integrates it as a component of the wave-propagation system, but the framework's core predictions regarding CSF medium disruption do not depend on the fascial argument being correct.

Fourth, the catecholamine-surge model is not wrong; it is incomplete. Catecholamine elevation after SAH is real and contributes to cardiac and systemic pathology. The wave-disruption model does not replace the catecholamine model; it subsumes it as one mechanism within a broader framework. The sympathetic surge itself may be a consequence of wave-coherence disruption in autonomic coupling pathways. Whether this reframing adds explanatory and predictive value beyond the standard model is an empirical question that the proposed tests can address.

Fifth, several of the proposed testable predictions involve human-subjects research (CSF acoustic measurements from ventriculostomy samples, serial imaging correlation with systemic outcomes, and ICP waveform analysis). These studies would require institutional review board approval and informed consent. The in vitro nerve-preparation experiments proposed in the anesthesia connection would provide an alternative approach to testing core aspects of the framework without human-subjects considerations.

## Conclusions

The human body may be usefully modeled as a wave system. We measure waves in every organ, define health by organized wave characteristics, and diagnose pathology by wave disruption. The composite homeostatic wave framework elevates this clinical observation to a theoretical principle: physiological homeostasis is the organized coherence of coupled oscillatory modes, and pathology results from disruption of this coherence through alteration of the propagation system--the fluid medium, the membrane substrates, or the coupling pathways. Aneurysmal SAH provides a dramatic natural experiment in wave-system disruption, in which blood entering the subarachnoid space simultaneously alters the acoustic impedance of the CSF medium and directly damages the leptomeningeal membrane network, a continuous collagenous structure with electrical properties that extends from the cranial meninges through the peripheral fascial system.

The loss of consciousness, multi-organ dysfunction, and persistent circadian and emotional sequelae are consistent with the disruption of a coupled oscillatory system through alteration of its propagation components. The soliton model and fascial signaling hypothesis provide mechanistic foundations, the CSD literature provides convergent evidence, and the five testable predictions outlined here are designed to distinguish this framework from the standard catecholamine-surge model. Should these predictions be confirmed, they would open new therapeutic avenues focused on restoring the propagation environment rather than solely managing downstream consequences. The framework additionally proposes that CSD--already recognized as a major contributor to delayed cerebral ischemia--may derive its pathological character specifically from medium disruption: the same altered CSF environment that drives global wave-coherence failure determines whether CSD triggers the vasoconstriction rather than vasodilation that converts reversible depolarization into tissue infarction, a distinction with direct implications for CSD-targeted therapeutic strategies.
